# HIV-1 integrase strand-transfer inhibitor resistance in southern Taiwan

**DOI:** 10.18632/oncotarget.24837

**Published:** 2018-05-18

**Authors:** Hung-Chin Tsai, I-Tzu Chen, Kuan-Sheng Wu, Yu-Ting Tseng, Cheng-Len Sy, Jui-Kuang Chen, Shin-Jung Susan Lee, Yao-Shen Chen

**Affiliations:** ^1^ Division of Infectious Diseases, Department of Medicine, Kaohsiung Veterans General Hospital, Kaohsiung, Taiwan; ^2^ Faculty of Medicine, School of Medicine, National Yang-Ming University, Taipei, Taiwan; ^3^ Department of Parasitology, Kaohsiung Medical University, Kaohsiung, Taiwan

**Keywords:** HIV, treatment naïve, drug resistance

## Abstract

The use of antiretroviral therapy has reduced rates of mortality and morbidity in patients with human immunodeficiency virus/acquired immune deficiency syndrome(HIV/AIDS). However, transmission of drug-resistant strains poses a challenge to control the spread of HIV-1. Primary resistance to integrase strand-transfer inhibitors (INSTIs) is rare despite their increased use. The prevalence of transmitted drug resistance (TDR) to INSTIs was 0.9% in northern Taiwan. This study was to analyse the prevalence and risk factors of TDR to INSTIs in southern Taiwan. In this study, we enrolled antiretroviral treatment-naïve HIV-1-infected subjects who underwent voluntary counselling and testing from 2013 to 2016 in southern Taiwan. Genotypic drug resistance, coreceptor tropism (CRT) and INSTI resistance were determined. Logistic regression was used to analyse the risk factors for INSTI polymorphic substitution. Sequences were obtained from 184 consecutive individuals, of whom 96.7% were men who have sex with men and 3.3% were heterosexual. Of the patients, 10% (19/183) had hepatitis B and 33.3% (61/183) had syphilis infection. Subtype B HIV-1 strains were found in 96.1% of the patients. Fifteen patients (8.4%, 15/178) harboured nucleoside reverse transcriptase inhibitors, non-nucleoside reverse transcriptase inhibitors or protease inhibitors resistance. CCR-5 coreceptors were used by 71.4% (130/184) of the patients. None of the patients had INSTI resistance-associated mutations, however 16 patients had INSTI polymorphic substitutions, and they were associated with a higher HIV viral load (*p* = 0.03, OR 2.4, CI 1.1–5.3) and syphilis infection (*p* = 0.03, OR 3.7, CI 1.1–12.0). In conclusion, no signature INSTI resistance-associated mutations were detected in our cohort. Continued monitoring of TDR to INSTI is needed due to the increased use of INSTIs.

## INTRODUCTION

Transmitted drug resistance (TDR) can reduce the treatment options available to newly HIV-1 infected patients, and it is associated with an increased risk of virological failure [1]. Surveillance of TDR in Taiwan has revealed the impact of improved antiretroviral therapy (ART) regimens, although routine pre-treatment genotypic drug resistance testing (GRT) in ART-naïve patients is not conducted in Taiwan due to financial constraints. Previous studies have reported prevalence rates of TDR for protease (PR)/reverse transcriptase (RT) (*pol* gene) of 8–11.1% [2, 3] in northern Taiwan and 10.6% in southern Taiwan [4].

The surveillance of TDR to integrase strand-transfer inhibitors (INSTIs), a relatively new drug class approved for the treatment of HIV, has become increasingly important in recent years [5, 6]. The integrase inhibitors raltegravir, dolutegravir and elvitegravir are increasingly used as first-line ART in combination with two nucleoside reverse transcriptase inhibitors (NRTIs) [5, 6]. Raltegravir was approved for clinical use in ART-experienced patients in 2009, and in 2012 it was approved for use in ARV-naive patients when used in combination with two NRTIs. Dolutegravir was approved as a first-line single tablet regimen (coformulated with abacavir/lamivudine) in June 2016 for treatment-naïve HIV-1 infected patients in Taiwan.

With the increasing use of INSTIs and subsequently treatment failure to INSTIs, the risk of TDR to INSTIs is expected to increase, especially in treatment-experienced patients who are not receiving suppressive antiretroviral drugs [7, 8]. However, despite the increasing use of INSTIs, few cases of transmission of INSTI resistance in treatment-naïve HIV-1 infected patients were reported in the SPREAD study [5] and northern Taiwan [3, 8]. Minor resistance mutations or polymorphisms have been reported to occur more often in non-B subtype HIV infections compared to subtype B infections [9, 10]. The main aim of this study was to analyse the prevalence of TDR to INSTIs in HIV-1 infected patients recruited from our voluntary counselling and testing (VCT) program in southern Taiwan, and to identify risk factors for its occurrence. The second aim of this study was to analyse the prevalence of TDR to PR/RT region and coreceptor tropism (CRT) in those patients.

## RESULTS

**Figure 1:**
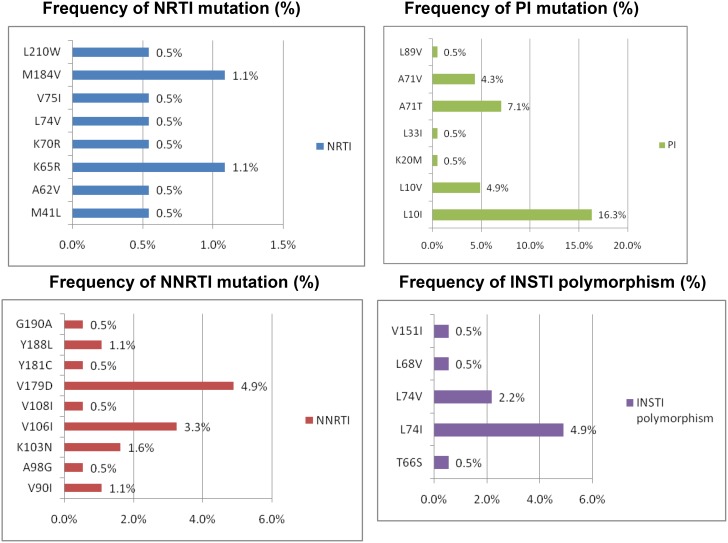
Percentage of HIV drug resistance-associated mutations to NRTIs, NNRTIs, PIs and INSTIs among 184 HIV-1-infected patients with GRT

From 2013 to 2016, a total of 184 patients were enrolled for GRT, all of whom were male with a medium age (IQR) of 26 (23–31) years. Of the 184 patients, 96.7% were men who have sex with men (MSM). The detailed demographic information was summaried at Table 1. The median CD4 cell count (IQR) was 308 (201–427) cells/μL, and the viral load (IQR) was 4.8 log (4.4–5.1). Most of the 184 patients (96.1%, 171/178) had HIV subtype B, and 3.9% (7/178) had CRF01_AE. The rate of successful sequencing was 96.7% (178/184) for PR/RT region and 100% for CRT and integrase region after repeating testing. Fifteen patients (8.4%, 15/178) harboured TDR to the *pol* (PR/RT) region (Table 1). Eight patients had resistance to NRTIs, 10 had resistance to NNRTIs, and only one had resistance to PIs. The most common drug resistance-associated mutations to NRTIs were M184V (1.1%) and K65R (1.1%), while those for NNRTIs were V179D (4.9%), V106I (3.3%), K103N (1.6%) and Y188L/V90I (1.1%) and those for PIs were L10I (16.3%), A71T (7.1%), and L10V (4.9%) (Figure 1). None of the 184 patients had TDR to INSTIs. However, 16 patients harboured virus with INSTI polymorphisms or substitution mutations, including L74I (4.9%), L74V (2.2%), T66 S (0.5%), V151I (0.5%), and L68V (0.5%) (Figure 1). In single variable analysis, those who had INSTI polymorphic substitutions wereassociated with a higher HIV viral load (IQR) (5.0 (4.7–5.3) vs. 4.8 (4.4–5.1), *p* = 0.02), positive hepatitis A antibodies (OR 4.0, confidence interval (CI) 1.1–14.2, *p* = 0.05) and syphilis infection (OR 3.3, CI 1.1–9.9, *p* = 0.04). In logistic regression analysis, the patients who had INSTI polymorphic substitutions were still associated with a higher HIV viral load (OR 2.4, CI 1.1–5.3, *p* = 0.03) and syphilis infection (OR 3.7, CI 1.1–12.0, *p* = 0.03). The INSTI polymorphic substitutions were not associated with risk factors for HIV acquisition according to the VCT questionnaires, HIV subtype, CD4, opportunistic infection markers, hepatitis B or C, *pol* (PR/RT) resistance or CRT.

**Table 1:** Demographic and resistance data among 184 antiretroviral treatment-naïve HIV-1-infected voluntary counselling and testing clients from 2013 to 2016 in southern Taiwan

					Univariate regression		Multivariate regression
	All patients, *N* = 184	INSTI polymorphic substitution, *N* = 16	No INSTI polymorphic substitution, *N* = 168	*p* value	Unadjusted OR (95% CI)	*p* value	Adjusted OR (95% CI)
Gender, *n* (%)							
Male	184 (100)	16 (100)	168 (100)				
Female	0 (0)	0 (0)	0 (0)				
Age (years), median (IQR)	26 (23–31)	25 (21–35)	26 (23–31)	0.47			
Route of transmission, *n* (%)							
MSM	176/182 (96.7)	15/15 (100)	161/167 (96.4)	1.00			
Heterosexual	6/182 (3.3)	0/15 (0)	6/167 (3.6)				
Duration of infection, *n* (%)							
Recent infection	92 (50)	9 (56)	83 (49)	0.80	1.3 (0.5–3.7)		
Chronic infection	92 (50)	7 (44)	85 (51)				
HIV VL (log), median (IQR)	4.8 (4.4–5.1)	5.0 (4.7–5.3)	4.8 (4.4–5.1)	0.02^*^	(0.1–0.8)	0.03^*^	2.4 (1.1–5.3)
CD4 count (cells/ul), median (IQR)	308 (201–427)	267 (240–426)	312 (200–430)	0.86			
HAV Ab, *n* (%)							
Positive	18/183 (9.8)	4/15 (26.7)	14/168 (8.3)	0.05^*^	4.0 (1.1–14.2)	0.09	3.5 (0.8–11.4)
HBs Ab, *n* (%)							
Positive	87/183 (47.5)	7/15 (46.7)	80/168 (47.6)	1.00			
HBs Ag, *n* (%)							
Positive	19/183 (10.4)	4/15 (26.7)	15/168 (8.9)	0.05		0.16	2.7 (0.7–10.8)
HBc Ab, *n* (%)							
Positive	45/183 (24.6)	5/15 (33.3)	40/168 (23.8)	0.53			
HCV Ab, *n* (%)							
Positive	4/183 (2.2)	0/15 (0)	4/168 (2.4)	1.00			
IHA-Amebiasis (≥256), *n* (%)							
Positive	4/162 (2.5)	0/15 (0)	4/147 (2.7)	1.00			
Toxoplasma-IgG, *n* (%)							
Positive	15/183 (8.2)	0/15 (0)	15/168 (8.9)	0.62			
CMV-IgG, *n* (%)							
Positive	179/182 (98.4)	15/15 (100)	164/167 (98.2)	1.00			
Cryptococcal Ag, *n* (%)							
Positive	0/183 (0)	0/15 (0)	0/168 (0)				
Syphilis, *n* (%)							
Positive	61/183 (33.3)	9/15 (60)	52/168 (31)	0.04^*^	3.3 (1.1–9.9)	0.03^*^	3.7 (1.1–12.0)
HIV subtype, *n* (%)							
Subtype B	171/178 (96.1)	16/16 (100)	155/162 (95.7)	1.00			
Non-subtype B (CRF01_AE)	7/178 (3.9)	0/16 (0)	7/162 (4.3)				
*pol* (PR/RT) resistance, *n* (%)							
Resistance	15/178 (8.4)	1/16 (6.2)	14/162 (8.6)	1.00			
Non-resistance	163/178 (91.6)	15/16 (93.8)	148/162 (91.4)				
*pol* (PR/RT) mutation, *n* (%)							
Mutation	78/178 (43.8)	10/16 (62.5)	68/162 (42)	0.19			
Non-mutation	100/178 (56.2)	6/16 (37.5)	94/162 (58)				
NRTI mutation	9/178 (5.1)	0/16 (0)	9/162 (5.6)	1.00			
NNRTI mutation	24/178 (13.5)	3/16 (18.8)	21/162 (13)	0.46			
PI mutation	57/178 (32)	8/16 (50)	49/162 (30.2)	0.16			
Tropism V3 receptor type, *n* (%)							
CCR5 type	131/184 (71.2)	13/16 (81.3)	118/168 (70.2)	0.56			
CXCR4 type	53/184 (28.8)	3/16 (18.8)	50/168 (29.8)				
INSTI resistance, *n* (%)							
Resistance	0/184 (0)	0/16 (0)	0/168 (0)				
Non-resistance	0/184 (0)	0/16 (0)	0/168 (0)				

INSTI: integrase strand-transfer inhibitors. OR:odds ratio. IQR: interquartile range. HAV Ab: hepatitis A antibody. HBs Ab: hepatitis B surface antibody.

HBs Ag: hepatitis B surface antigen. HBc Ab: hepatitis B core antibody. HCV Ab: hepatitis C virus antibody. IHA: Indirect Hemaggluuutination. CMV: cytomegalovirus. PR/RT: protease/ reverse transcriptase. NRTI: nucleoside reverse transcriptase inhibitor. NNRTI: non-nucleoside reverse transcriptase inhibitor. PI: protease inhibitor. CCR5: C-C chemokine receptor type 5. CXCR4: C-X-C chemokine receptor type 4. Recent infection was defined as recent high risk behavior with compatible retroviral symptoms 3 months before serological positivity; (2) a positive enzyme immunoassay test for HIV-1 with indeterminate Western blot results, and two separate positive results for reverse transcriptase-polymerase chain reaction; or (3) negative serologic tests 6 months ago [11].

**Figure 2:**
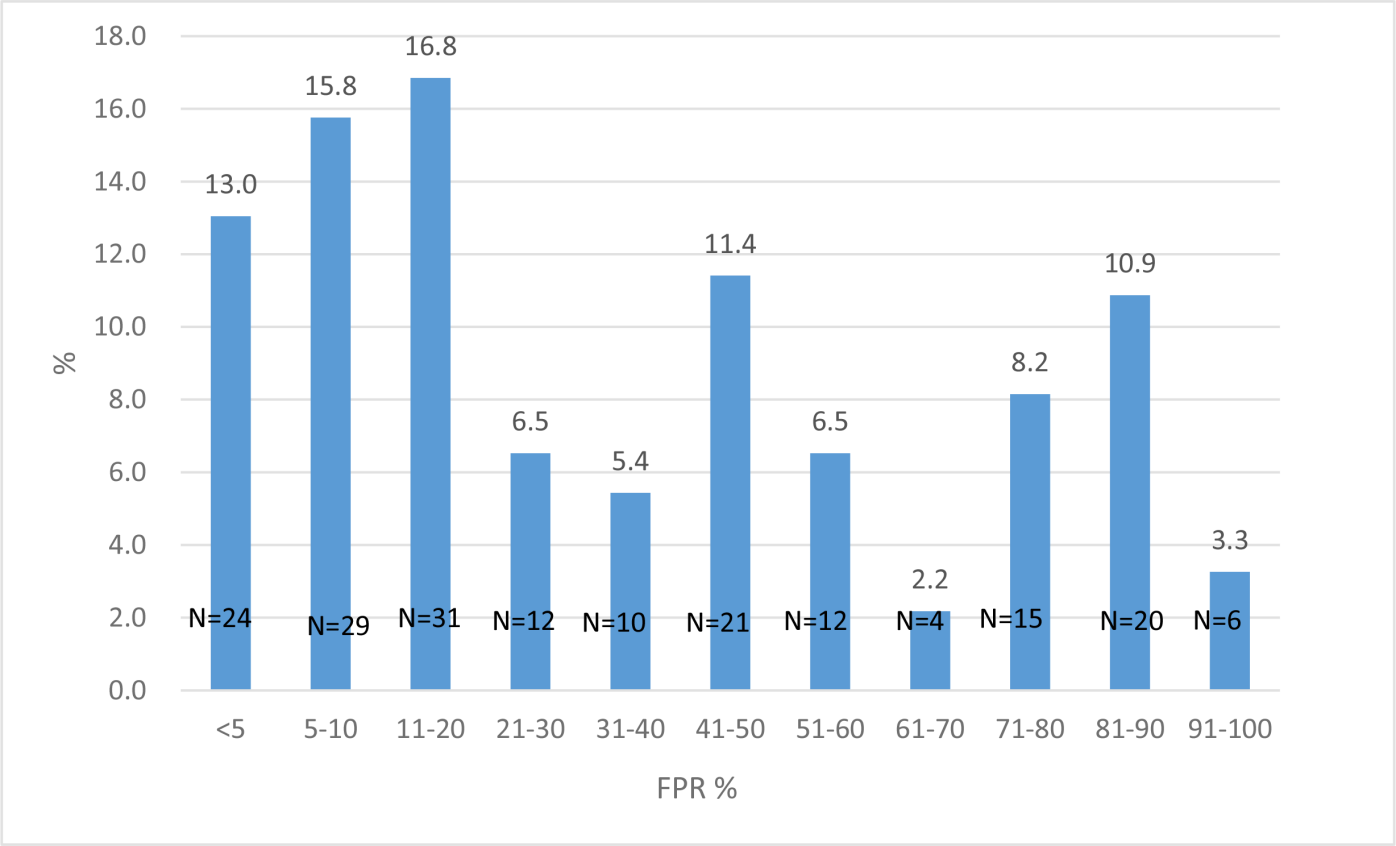
The distributions of false-positive rates (FPRs) for CRT among the 184 patients, of whom 71.2% had only R5-tropic strains (FPR 10%) Some of the CRT results (*n* = 108) had been reported in the previous study [13].

CRT predictions indicated that 71.2% (131/184) of the patients had only R5-tropic strains. There was no significant difference in the frequency of X4 viruses in single analysis or triplicate testing. The distribution of false-positive rates (%) among the 184 patients is shown in Figure 2. There were no significant associations between CD4 cell counts, risk factors for HIV acquisition, HIV subtype, opportunistic infection markers or TDR with the presence of R5-tropic viruses. All of the sequences were submitted to NCBI.

## DISCUSSION

The results of the present study show the prevalence of HIV TDR among HIV-infected treatment-naïve patients enrolled at our VCT service centre in southern Taiwan. In particular, the results highlight the moderate rate of resistance (8.4%) to NRTIs/NNRTIs/PIs, and the absence of any signature INSTI resistance mutations. However, INSTI polymorphic substitutions were not rare, and those who had INSTI polymorphic substitutions were more likely to have a higher HIV viral load and syphilis infection. The absence of INSTI resistance-associated mutations in this study is similar to the CORONET study [9], the Italian Cohort of Antiretroviral Naive study group [15], the National Taiwan University Hospital Study [8] and the epidemiological surveillance study of the European SPREAD programme in 2006–2007 [5], in which primary resistance to INSTIs was rare.

However, in the present study, INSTI polymorphic substitutions were detected in some of the patients (8.6%, 16/184), especially in those with a high viral load and syphilis infection. It is unclear whether these naturally occurring mutations impair fitness or compensate for other mutations [16, 17]. L74I was the mostly commonly found polymorphic substitution in our study, which is a less common mutation associated with secondary resistance to clinical INSTIs. It is believed that this mutation modulates the effects associated with major drug resistance, however further studies are needed to clarify its contribution to viral fitness and compensatory resistance mechanisms [16]. The effect of V151I on susceptibility in recombinant virus is also unclear, and may depend on the plasmid backbone used [16, 18, 19].

In the study of Meixenberger *et al*. [20], they found that those polymorphic substitutions could control enzymatic activity and replication capacity independent of selective pressure of INSTIs. Further more, the accessory drug resistance mutations that compensate viral fitness are often already polymorphic in drug-sensitive HIV-1, suggesting that these mutations may naturally enhance viral fitness and virulence [21, 22]. The high viral load in drug naïve HIV-1 infected patients was already found to be associated with treatment-associated polymorphism in protease [21]. It was possible that the increased polymorphic substitutions in our patients with high viral load might be related to enhance or compensate viral fitness due to retrovirus replication errors. The increased INSTI polymorphic substitutions in HIV-1 infected VCT with concurrent syphilis infection might be indirect to increase HIV plasma viral load. It was found that concurrent infection with syphilis in HIV infected individuals could increase the HIV plasma viral load [23]. Since 50% of the VCT clints were recent infection with HIV, and syphilis shared a same transmission route with HIV. It was possibly that recent infection with HIV increased the plasma viral load and futher syphilis coinfection exaggerated the highness of viral load. Further study was needed to clarify this phenomenon.

Our study could not be compared to those reported from northern Taiwan [3, 8]. In the study of Lai *et al.* [3], they reported that the prevalence of INSTI mutation was 3.2%. They used the Stanford HIV Drug Resistance Database for analyzing the integrase resistance mutations instead of IAS-USA drug resistance list in our study. In the study of Chang *et al.* [8], they reported the overall prevalence of major resistance mutations to INSTIs was 0.9% (*n* = 12) among the 1307 HIV-1 samples from patients never exposed to INSTIs. However, they enrolled those patients seeking for HIV care at their hopsital, not VCT clients like ours. Among the 11 patients with CD4 count less than 200 cells/ul, 36.5% (4/11) harbored INSTI resistant viruses. In their study population, the patient's age was older (33 y/o), 31.7% (405/1277) with CD4 count <200 cells/ul, and only 84.1% (1035/1230) was MSM. Although our study used the same INSTI resistance mutation intepretation standard with theirs, all of the differences made the comparsion between these 2 studies difficulities. Our study was epidemiologically important. Unlike the study in the metropolitan area in northern Taiwan [8]. Our study was the most comprehensive TDR study in VCT clients in southern Taiwan, involving not only the PR/RT, integrase region but also CRT.

Raltegravir was introduced into Taiwan in 2006, but it was not available for ARV-naive patients until 2012, when it was approved for use when combined with two NRTIs. Dolutegravir was introduced into Taiwan in 2010 when a few patients were enrolled in a clinical trial (SAILING). It was then made available as the preferred regimen for ARV-naive patients to be used as a single tablet regimen (ABC/3TC/DTG) in June 2016. Elvitegravir is not available in Taiwan. Raltegravir and elvitegravir have a low genetic barrier to resistance, and extensive overlap exists in their drug resistance profiles [24, 25]. Raltegravir is prescribed as first-line and salvage therapy for patients with drug-resistant HIV who may be prone to INSTI resistance, which could be transmitted to newly-infected patients. Dolutegravir has a higher genetic barrier to resistance than raltegravir and elvitegravir, however its long-term potency may be compromised in the presence of Q148R/H/K plus one or two additional mutations [24, 25]. As dolutegravir is the preferred regimen and salvage ART, dolutegravir resistance may evolve in Taiwanese patients in the future along with the increasing use of INSTI treatment for HIV-1 infection. Continued surveillance of INSTI resistance in Taiwan is thus warranted.

The prevalence of transmitted HIV resistance to NRTIs/NNRTIs/PIs in this study was around 8.4%, which is similar to a report conducted in northern Taiwan (8–11.1%) [2, 3]. However, routine pre-treatment GRT in ART-naïve patients is lacking in Taiwan because of financial constraints. The prevalence of TDR has recently increased to 5% in some areas of South Africa, Kenya, and Zambia, and up to 15% in Uganda [26–28]. In Asia, this rate is around 4–12%, including 3.8% in China [29], 7.7% in Japan [30], 12% in South Korea [31], and 4.9% in Thailand [32].

Previous studies in which HIV-1 subtype B is the predominant strain have reported that 80–90% of HIV-1-infected treatment naïve individuals harbour R5 viruses [33]. A Spanish study reported that 13.4% of 67 HIV-1 seroconverters harboured CXCR4 viruses [34], and a French study reported that 15.9% of 390 patients with primary HIV infection subtype B had X4 viruses [35]. In the current study, only 71% of the treatment-naïve patients had R5-tropic virus. This finding is similar to the study of Meini *et al.* [36], who reported that 26.2% of HIV-infected treatment-naïve patients in Italy had non-CCR5-tropic viruses. This discrepancy in the prevalence of CRT in different studies may be due to different patient populations, stage of HIV infection (primary vs. chronic infection) and methodology of CRT. Studies such as ours based on genotypic methods have generally reported a higher prevalence of CXCR4.

Therefore, it seems as though integrase genotyping is not necessary before initiating INSTI therapy, as long as transmitted INSTI resistance remains at a low level. However, it is essential to perform integrase gene resistance testing in subjects who fail raltegravir therapy, as cross-resistance to dolutegravir is common [24], and patients should not be kept on a failing INSTI regimen.

There are several limitations to this study. First, it was based on patients enrolled from a VCT program in southern Taiwan. These patients had high CD4 counts and the majority were MSM. MSM is the leading risk behaviour for HIV infection, and TDR is more prevalent among MSM in Taiwan. Therefore, the results of this study may not be generalized to intravenous drug abuser and heterosexual populations. Second, the number of HIV-1-infected patients was relatively small compared to those of other studies. Third, we did not perform detuned ELISA HIV antibody test to differentiate our VCT patients with recent or chronic infection. The results would be more reliable compared to just based on the patient's history, western blot and clinical manifestations. Finally, the prevalence of INSTI resistance-associated mutations may be underestimated due to the low sensitivity of population sequences compared to next generation sequencing.

In conclusion, no INSTI-resistant variants were detected in our VCT clients with HIV-1 infection. However, polymorphic substitutions were not rare (8.7%) and associated with high viral load and syphilis infection. As the use of INSTIs becomes more widespread, continued surveillance of primary INSTI resistance is warranted.

## PATIENTS AND METHODS

### Ethical statement

This study was approved by the Institutional Review Board of Kaohsiung Veterans General Hospital, Taiwan (VGHKS98-CT1-08, VGHKS13-CT4-12 and VGHKS15-CT5-10). The protocol complied with all ethical considerations involving human subjects, and all information obtained followed standard clinical guidelines. All of the study participants provided informed consent.

### Study setting and participants

Plasma samples were collected from a consecutive group of individuals recruited from our free VCT center at Kaohsiung Veterans General Hospital from January 2013 to December 2016 (*n* = 184). In Taiwan, free VCT services at the point of delivery were established in 1996 and are sponsored by the Taiwan Center for Disease Control (CDC) as a key strategy to promote the early diagnosis and prevention of HIV and other sexually transmitted diseases (STDs), and to encourage referral to treatment. The VCT procedure included a 30-minute session of integrated pre-test and post-test counselling, followed by collection of a 5–10 mL blood sample for serological testing for HIV infection and syphilis. If the clients had reactive HIV ELISA or positive rapid test results, they were referred for clinical evaluation, Western blot examination and treatment.

Recent infection was defined as recent high risk behavior with compatible retroviral symptoms 3 months before serological positivity; a positive enzyme immunoassay test for HIV-1 with indeterminate Western blot results, and two separate positive results for reverse transcriptase-polymerase chain reaction; or negative serologic tests 6 months ago [11].

Due to financial constraints on the provision of free access to combination antiretroviral therapy (cART), the Taiwan CDC limits the prescription of cART to antiretroviral-naive HIV-1-infected patients who received their first cART after 1 June 2012. The following laboratory examinations were performed when the clients returned to the clinic: CD4 cell count (FACS Flow, Becton Dickinson and Company, Franklin Lakes, New Jersey, USA), plasma viral load (Cobas Amplicor HIV-1 monitor test, V.1.5. Roche Diagnostics Corporation, Indianapolis, Indiana, USA), serological markers for syphilis, hepatitis A, B and C, cryptococcus, toxoplasmosis, cytomegalovirus and amoebiasis, and cortisol level and liver, renal and thyroid function.

### Genotypic drug resistance testing and coreceptor tropism

Resistance testing for PR/RT (*pol* gene) was performed on plasma samples using a ViroSeq HIV-1 Genotyping System version 2.8, according to the manufacturer's instructions (Celera, Alameda, CA, USA). INSTI resistance and coreceptor tropism were determined using in-house population sequencing [12, 13]. Antiretroviral resistance to PR/RT (*pol* gene) and INSTI was interpreted using the HIVdb program of the Stanford University HIV Drug Resistance Database (http://hivdb.stanford.edu; date last accessed, 10 December 2016). The patients classified as having low-level resistance, intermediate resistance and high-level resistance were defined as having drug resistance. Resistance-associated mutations were defined by the presence of at least one mutation included in the 2017 drug resistance mutation list of the International AIDS Society-USA consensus guidelines [14]. INSTI polymorphic substitutions were defined as mutations not defined as a major INSTI mutation (Y143C/H/R, Q148H/K/R, N155H) and not belonging to the integrase substitutions with a Stanford HIVdb score ≥10 to at least one INSTI (H51Y, T66A/I/K, L74M, E92G/Q/V, Q95K, T97A, F121Y, E138A/K, G140S/C/A, Y143G/K/S/A, P145S, Q146P, S147G, V151A/L, S153F/Y, N155S/T, E157Q, G163 K/R, S230R, and R263K) [8].

The coreceptor tropism of all samples was predicted by using the geno2pheno_[coreceptor]_ service at the following web site: http://coreceptor.bioinf.mpi-inf.mpg.de/index.php. All of the samples were run in triplicate, and the sequence prediction results above a false-positive rate of 10% were considered to be CCR5 tropic. Those at or below a false-positive rate of 10% were considered to be CXCR4 or D/M tropic [13].

### Statistical analysis

The Mann-Whitney *U* test was used to compare the median values of continuous variables between groups (resistance/polymorphic substitution and wild virus), while Fisher's exact test was used to compare categorical variables between the two groups. A two-sided *p* value < 0.05 was considered to be statistically significant. Logistic regression analysis was used to determine the factors associated with INSTI-associated resistance or polymorphisms. Variables with a *p* value of < 0.1 in univariate analysis were included in the logistic regression model. ORs and its 95% CIs were estimated. All statistical analyses were performed using SPSS software version 18.0 (SPSS Inc., Chicago, Illinois, USA).

### Transparency declarations

None to declare.
